# Supplementary material for: MetaRibo-Seq measures translation in microbiomes
Source: Nat Commun. 2020 Jun 29;11:3268. doi: 10.1038/s41467-020-17081-z (PMC7324362; doi:10.1038/s41467-020-17081-z)
Supplement: Supplementary file 10 — Supplementary Data 7 [file 41467_2020_17081_MOESM10_ESM.zip › File2/Confidence_High_Taxonomy/83621_out.krona.html]

Javascript must be enabled to view this page.

members
magnitude
magnitudeUnassigned
count
unassigned
taxon
rank

83621\_out

24

24
superkingdom
2

1239
phylum
24

class
24
186801

24
order
186802

family
23
31979

1485
23
genus

species
23

SRS011405\_contig\_number\_16764SRS013940\_contig\_number\_contig-100\_407.133502SRS017103\_contig\_number\_40598SRS017433\_contig\_number\_16546SRS020233\_contig\_number\_contig-100\_80.342427SRS020869\_contig\_number\_234SRS023526\_contig\_number\_39401SRS024009\_contig\_number\_8101SRS042628\_contig\_number\_12765SRS054905\_contig\_number\_14386SRS062701\_contig\_number\_contig-100\_2.70093SRS063190\_contig\_number\_14257SRS076756\_contig\_number\_22456SRS103987\_contig\_number\_7960SRS1041142\_contig\_number\_6305SRS1041145\_contig\_number\_contig-100\_44.196062SRS142781\_contig\_number\_contig-100\_75.90648SRS147346\_contig\_number\_68684SRS147977\_contig\_number\_contig-100\_91.91859SRS148424\_contig\_number\_7778SRS148874\_contig\_number\_contig-100\_1682.105061SRS893170\_contig\_number\_9853SRS893378\_contig\_number\_18803
59620

186803
family
1

1
genus
830

species
1

SRS104693\_contig\_number\_13336
28121
